# Challenges in water kefir production and limitations in human consumption: A comprehensive review of current knowledge

**DOI:** 10.1016/j.heliyon.2024.e33501

**Published:** 2024-06-22

**Authors:** Eda Bozkir, Birsen Yilmaz, Heena Sharma, Tuba Esatbeyoglu, Fatih Ozogul

**Affiliations:** a Department of Agricultural, Food and Environmental Sciences, Marche Polytechnic University, Italy; b Department of Biological Sciences, Tata Institute of Fundamental Research, Hyderabad, India; c Department of Nutrition and Dietetics, Faculty of Health Sciences, Cukurova University, 01330, Adana, Turkiye; d Food Technology Lab, Dairy Technology Division, ICAR-National Dairy Research Institute, Karnal, Haryana, India; e Department of Molecular Food Chemistry and Food Development, Institute of Food and One Health, Gottfried Wilhelm Leibniz University Hannover, Am Kleinen Felde 30, 30167, Hannover, Germany; f Department of Seafood Processing Technology, Faculty of Fisheries, Cukurova University, 01330, Adana, Turkiye; g Biotechnology Research and Application Center, Cukurova University, Adana, 01330, Turkiye

**Keywords:** Water kefir, Fermentation, Health benefits, Microbial diversity, Lactic acid bacteria

## Abstract

Water kefir is a convenient dairy-free alternative to dairy-based fermented beverages. It is prepared by fermenting a sucrose solution with fresh and dried fruits using water kefir grains, and demineralized whey can be used in water kefir production. This review describes current knowledge on water kefir production and its health effects. The main aims of this paper are to focus on the microbial composition, potential health-promoting properties, limitations in human consumption, and challenges in the production of water kefir. Water kefir grains and substrates, including brown sugar, dried and fresh fruits, vegetables, and molasses, used in the production influence the fermentation characteristics and composition of water kefir. Lactic acid bacteria, acetic acid bacteria, and yeasts are the microorganisms involved in the fermentation process. *Lactobacillus* species are the most common microorganisms found in water kefir. Water kefir contains various bioactive compounds that have potential health benefits. Water kefir may inhibit the growth of certain pathogenic microorganisms and food spoilage bacteria, resulting in various health-promoting properties, including immunomodulatory, antihypertensive, anti-inflammatory, anti-ulcerogenic, antiobesity, hypolipidemic, and hepatoprotective activities.

## 1 Introduction

Water kefir has a hazy and sticky consistency, a blond to yellowish color, an even, organized, bubbling texture, an acidic (about 2 % lactic acid content) and yeasty flavor (less than 1 % alcohol), with a tinge of sweetness. It is a locally or homemade fermented beverage produced from sucrose solution incorporated with fresh and dried fruits along with water kefir grains. Little data is available on the origin of water kefir, although the first report on water kefir grains dates back to the 1890s, when the “ginger-beer plant” (originated from the Caucasus region) was introduced in Britain by soldiers returning from Crimea. It is significantly different from typical milk kefir, which is produced from bovine milk fermented with a defined set of cultures and possesses distinct physicochemical and microbiological properties [1,2]. After sifting the finished product, a procedure known as "pitching"- the water kefir grains are used again for subsequent fermentation. The method is known as "back-slopping" if some of the fermented beverage is added to a new batch of the fermentation process (in addition to the grains). The optimum temperature of fermentation varies between 20 and 25 °C for 24–72 h wherein, usually 6–10 % sucrose and 6–20 % grains are used [3]. Water kefir produced from *Aronia melanocarpa* juice and pomace demonstrated immense potential in water kefir production with high total phenolic content, total flavonoid and anthocyanin content, and acceptable sensory attributes [4]. The other sources used for water kefir production include demineralized whey along with Dimrit and Shiraz Grape varieties [5]. The source of sugar plays an important role in the production of water kefir, and raw sugar cane along with dried figs are generally used as a base for fermentation. Generally, the microorganisms used for the production of water kefir grains belong to lactic acid bacteria (LAB), acetic acid bacteria (AAB), and yeasts, and fermentation brought about by water kefir grains is spontaneous. However, a few reports have identified the strains of *Lentilactobacillus hilgardii* that produce dextran (α-(1 → 6)-linked glucose polymer) forming granules. Glycosyltransferase is mainly responsible for dextran production [6].

Water kefir production is a storehouse of a diverse range of microorganisms involved in fermentation. There have been reports on the isolation and draft genome sequencing of *Oenococcus* *kitaharae* CRBO2176 from homemade kefir prepared from figs [7]. In addition, the diversity of microorganisms present in water kefir and the challenges associated with the production of water kefir, including the source of sugar, time-temperature combination for fermentation and production up-scaling, and its significant bioactive compounds, including polypeptide, polysaccharide, organic acid, and other compounds, generated during fermentation make it a significant part of the human diet and play a pivotal role in health improvement and maintenance [8]. Such beverages are most suitable for people who do not wish to consume animal-origin products; as water kefir offers a viable substitute for dairy-fermented beverages. Owing to acid production, the growth of certain pathogenic microorganisms, including *Salmonella* sp., *Shigella* sp., and *Staphylococcus* sp., is inhibited. Several reports have shown the immunomodulatory, antihypertensive, anti-inflammatory, anti-ulcerogenic, hypolipidemic, and hepatoprotective activities of water kefir consumption [[9], [10], [11]]. Considering the advances in water kefir production associated with microbial diversity and health benefits, the present review aims to explore the microbial composition and technological properties of water kefir, the limitations of water kefir for human consumption, the challenges in water kefir production, and the potential health-promoting effects of water kefir.

## 2 Microbial diversity of water kefir

The steps of typical water kefir and milk kefir production are somewhat similar; however, the microbial and chemical composition of water kefir is quite different from that of milk kefir. Water kefir grains and substrates such as brown sugar, dried and fresh fruits, vegetables, and molasses, which are used in the production of water kefir, influence the fermentation characteristics and composition of water kefir [8]. Milk kefir grains, like water kefir grains, are assemblages of the same major microbial groups in a polysaccharide matrix. Nevertheless, milk kefir grains can contain species that are not found in water kefir, such as *Lb. kefiranofaciens*, which is responsible for producing the heteropolysaccharide kefiran. Nonetheless, *Lentilactobacillus hilgardii* produces a homopolysaccharide, dextran, which is the predominant exopolysaccharide of water kefir grains and water kefir beverages [12,13]. A study in which water kefir grains were compared with milk kefir grains found a significant difference in terms of microbiological content, chemical properties, and color of the grains (*p* < 0.05) [14].

Many studies have reported the microbial composition of water kefir (also known as sugar/sugary kefir), and all of them reported the presence of *Lactobacillus* species (Table 1). According to these studies, the most common species in water kefir are *L. hilgardii*, *L. nagelii, L. casei,* and *L. paracasei, whereas L. hilgardii* and *L. nagelii* are the main *Lactobacillus* species in water kefir grain [12]. In addition to LAB, water kefir grains and beverages contain yeasts such as *Saccharomyces cerevisiae*, bifidobacterial such as *Bifidobacterium aquikefiri*, and AAB such as *Acetobacter fabarum* [13]. Water kefir was fermented at 20 °C for 72 h, and the minimum and maximum levels of the microbial groups were characterized. The maximum and minimum levels of LAB, AAB, and yeast were determined as 9.0 × 10^7^ CFU/mL vs 2.8 × 10^4^ CFU/mL, 3.2 × 10^6^ CFU/mL vs 7.0 × 10^2^ CFU/mL and 4.8 × 10^7^ CFU/mL vs 4.77 × 10^5^ CFU/mL, respectively [6]. In a previous study, several LAB species (*Liquorilactobacillus nagelii*, *Lentilactobacillus hilgardii*, *Lentilactobacillus farraginis*, *Liquorilactobacillus satsumensis*, *Schleiferilactobacillus harbinensis*, *Lentilactobacillus diolovorans*, and *Lacticaseibacillus casei/paracasei*), AAB species (*Acetobacter tropicalis*, *Acetobacter indonesiensis*, *Acetobacter lovaniensis*), and *Gluconobacter oxydans* isolated from water kefir samples [15].

**Table 1:** Microbial composition of water kefir beverages.

Origin	Substrates and fermentation	Technique	Main species	Reference
Malaysia	− 18 g WKG, 50 g organic raw sugar and 500 mL of mineral water. − 24 h at room temperature	16S rRNA sequencing	*Lb. hilgardii, Lb. harbinensis, Acetobacter lovaniensis, Lb. satsumensis, Acetobacter tropicalis, Lb. zeae, Oenococcus oeni*	[16]
Argentina	− 10 g WKG, 100 mL sugar solution - Three sugar solutions; 5 % (w/v) brown sugar, 6.5 % (w/v) purified molasses and 6.5 % (w/v) high-test molasses	16S rRNA sequencing	Brown sugar and purified molasses: *Lb. nagelii* High-test molasses: *Lb. hilgardii/diolivorans* and *Lb. casei/paracasei*	[17]
Japan	− 10 g WKG, 100 mL sugar solution - Three sugar solutions; 5 % (w/v) brown sugar, 6.5 % (w/v) purified molasses and 6.5 % (w/v) high-test molasses − 24 h at room temperature	16S rRNA sequencing	Brown sugar: *Lb. nagelii, S. cerevisiae, A.tropicalis* Purified molasses: *A. tropicalis, Lb. farraginis, Lb. satsumensis, Oenococcus kitaharae, Lb. casei/paracasei, Lb. nagelii, Lb. harbinensis, Pichia occidentali, S. cerevisiae* High-test molasses: A. Indonesiensis, *Lb. casei/paracasei, Lb. diolivorans, Lb. farraginis, Lb. harbinensis, Pichia membranifaciens, S. cerevisiae* Species isolated from kefir beverage for the first time: *Acetobacter indonesiensis, Acetobacter tropicalis, Gluconobacter oxydans*, *Lb. farraginis*, *Oenococcus kitaharae*, and *Pichia occidentalis*	[15]
Belgium	- Unrefined cane sugar (7.1 %, m/v) and fig extract (17.6 %, v/v) − 15 g WKG, 85 mL water kefir simulation medium	Shotgun metagenomic sequencing	*Bifidobacterium aquikefiri, Lb. harbinensis, Lb. hilgardii, Lb. nagelii, Lb. paracasei, Oenococcus oeni, Oenococcus kitaharae* *Saccharomyces cerevisiae, Dekkera bruxellensis,*	[13]
Canada, the United Kingdom, the United States	− 60 g grains per L of sterilised mineral water − 10 % sucrose, one dried, organic fig − 24 h at room temperature	Culture-independent	- *Zymomonas* was the dominant component followed by lactic acid bacteria (*Lactobacillus* and *Leuconostoc*) and acetic acid bacteria (*Acetobacter* and *Gluconacetobacter*). - *Bifidobacteriaceae* was at low proportions.	[18]
Germany	- Four different home-made grains - Mineral with 100g/L fig extract and 80 g/L sucrose − 72 h at 21 °C	16S rRNA sequencing	*Lactobacillus, Leuconostoc, Acetobacter, Gluconobacter, Bifidobacterium* (*Bifidobacterium psychraerophilum*)	[19]
Turkey	- WKG 5 % (*w/w*) added to Rosehip, Cornelian cherry, pomegranate, red plum, and hawthorn juices − 48 h at room Temperature	Culture dependent	- Rosehip kefir: highest *Lactobacillus* spp., and *Lactococcus* spp. - Cornelian Cherry and hawthorn kefir: yeast counts increased	[20]
Belgium	− 15 g WKG, 85 mL of autoclaved water kefir simulation medium − 6 g unrefined cane sugar, 65 mL of distilled water and 20 mL of fig extract	Culture-independent	*Lb. paracasei, Lb. hilgardii,* *Lb. nagelii* and *S. cerevisiae*	[21]
Germany	- Homemade WKGs − 1 L tap water, sucrose solution (100 g/L), two dry figs and a slice of organic lemon − 72 h at 21 °C	16S rRNA sequencing	*Lb. hordei, Lb. nagelii, Lc. mesenteroides, Lc. citreum, Acetobacter fabarum, Acetobacter Orientalis, S. cerevisiae, Lachancea fermentati, Hanseniaspora valbyensis, Zygotorulaspora florentina*	[22]

16S rDNA: 16S ribosomal RNA gene; WKG: water kefir grain.

The microbial composition, chemical and sensory characteristics of water kefir can vary and these parameters may influence the quality of water kefir. It was found that different sugar solutions (brown sugar, purified molasses, and high-test molasses) affected the microbiota of water kefir during multiple fermentation of kefir [17]. Except for high-test molasses beverage, which was dominated by *Proteobacteria* (up to 78 % of the total population and mainly *Acetobacter lovaniensis* and *Gluconobacter oxydans/roseus*) and *Firmicutes* (main species were *Liquorilactobacillus nagelii, Lentilactobacillus hilgardii/diolivorans* and *Lacticaseibacillus casei/paracasei*) were the main microbial group in water grains and beverages made from different sugar substrates (up to 98 % of the total population) [17]. Similarly, a culture-dependent analysis showed that different bacterial successions might be taking place in three different sugar substrates (brown sugar, purified molasses, and high-test molasses) throughout seven cycles of repeated inoculation [15]. Another study also showed that using different types of sugar which were grape molasses, honey and unrefined sugar resulted in higher content of *Lactococcus* spp. and yeast in water kefir samples [23]. A study was conducted to investigate the microbial and chemical makeup of a water kefir fermentation and its associated microbial community by combining culture-dependent techniques, compositional metagenomics, and untargeted metabolomics [24]. The study found that the microbial community shifts over time throughout the fermentation process, with an enrichment of microbial groups after 72 h of fermentation. The dominant genera for LAB and AAB, according to metataxonomics results, are Lactobacillus and Acetobacter, while *P. membranifaciens* is the dominant species among yeast. The production of bioactive compounds has been shown using metabolomic analysis. Isoschaftosides, which are known for their anti-inflammatory and hepatoprotective activities were found in the matrix, which has not been reported before in the matrix [24]. Combining chemical data, cultured species, and microbial biodiversity data seem to be helpful in identifying key points in the fermentation process of food and understand the patterns of microbial populations involved. Another recent study compared the bacterial and fungal diversity of kefir beverages produced using milk or sugared water (water kefir) as fermentation matrices [25]. The results of comparative metatranscriptome analysis showed that the bacterial communities in all beverages were composed of Lactobacillaceae and Acetobacteraceae, but the yeast families differed between milk and water kefir. Saccharomycetaceae was more abundant in water kefir, while Dipodascaceae and Pichiaceae were more abundant in milk. The researchers also found that the kefir samples shared 70 % of functional orthologs for yeast genes but only 36 % for bacterial genes. This suggests that the yeast communities in the different kefir beverages have more similar functions than the bacterial communities [25]. These results supported that the microbiota of water kefir can change as per the substrates such as sugar type. Therefore, unsuitable substrates may cause to reduce the amount of microorganisms which have beneficial effects on human health. In addition, the question regarding the number of live microorganisms in grains following repeated fermentations to produce water kefir is another major issue that should be taken into consideration.

The grains which are used to obtain water kefir may also contribute to microbial diversity. Recently, traditional milk kefir grains have been used to produce water kefir-like beverages. It has been shown that the final products did not show the same abiotic and biotic composition as water kefir. Water kefir-like beverages had a lower amount of lactic acid and its counts, however, had higher ethanol concentrations than milk kefir. Besides, all beverages passed the sensory test and were deemed suitable for ingestion [26]. In contrast, it should be noted that because of the techniques that are used for the isolation of the microbial species, the results of the studies may show differences between each other. Culture-dependent and culture-independent techniques (which is amplicon sequencing of the V1–V4 and V4–V5 regions of the 16S rRNA gene using metagenomic DNA) are the main approaches to investigating microbial species present in water kefir and kefir grains [13,19]. According to the culture-dependent method, the main microbial populations of water kefir are *Lactobacillus, Lactococcus, Leuconostoc, Acetobacter, Saccharomyces, Hanseniaspora/Kloeckera, Zygotorulaspora* and *Candida.* Four kefir grains from different countries were used to produce water kefir and it has been found that the dominant bacteria were *Zymomonas* through the culture-independent method [18]. *Lb. hilgardii* and Lb. nagelii were identified by the culture-independent method, however, they were not detected by the culture-dependent method [27]. These results emphasize that the culture-independent method has more advantages than the culture-dependent. In addition, the fact that some bacteria are slow-growing or fastidious makes their identification difficult and both time- and resource-intensive. This is one of the major constraints of culture methods. Therefore, next-generation sequencing is suggested since it offers insight into the entire microbiome by enabling culture-free identification of a theoretically infinite number of diseases [28].

In a recent study, in order to increase the postbiotic composition of the fermented products, co-fermentation of symbiotic culture of bacteria and yeasts (SCOBY) and water kefir grains was developed, which was then further examined [29]. The antimicrobial activity against *Aspergillus niger* MIUG M5, *Staphylococcus aureus* ATCC 25923, *Escherichia coli* ATCC 25922, and *Bacillus subtilis* MIUG B1 was assessed, together with the total acidity, pH, and antioxidant capacity. The yeasts and bacteria from the water kefir grains and SCOBY microbiomes could coexist in symbiosis [29].

## 3 Technological properties of water kefir

**Fig. 1:**
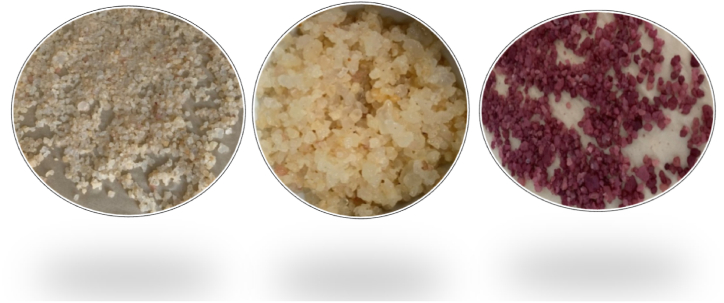
Water kefir grains. A: Dried water kefir grain, B: Water kefir grains used for the production of apples-based water kefir, C: Dried water kefir grains used for the production of *Aronia melanocarpa* juice-based water kefir (own pictures).

Water kefir, which is called also as sugar kefir and tibicos, is a sucrose-based, refreshing but slightly sweet and self-carbonated fermented beverage [[30], [31], [32]]. Even though the origin of the water kefir grain is not certain, water kefir grains are formed as granules that are fermented from the sap on the pads of the *Opuntia* cactus in Mexico according to the latest literature [30]. Fig. 1 shows the different granules of water kefir grains.

Kefir grains can be observed in a gelatinous and elastic form which is similar to cauliflower [33]. However, their appearance can also be described as “rock salt”, having a colour of whitish to grey [34]. The grains contain almost 45 % mucopolysaccharide, 34 % protein, and 4 % fat together with the group B vitamins, vitamin K, calcium, phosphorous, and magnesium [33].

Physically, the grains are composed of a peripheral part and an inner part. The peripheral part consists of bacteria, while the inner part contains mainly yeast. Among these two parts, bacteria and yeasts have long polysaccharide filaments. Through refrigeration, lyophilizing, vacuum drying, and freezing, the grains can be conserved for the next fermentation. If they are stored under optimal conditions, grains can never be damaged [33]. In this symbiotic system, yeasts serve as a source of nitrogen that can be used by bacteria for assimilation [8].

The traditional production of water kefir mostly occurs on a small scale and there is not enough data neither to investigate the surrounding conditions or the starter cultures [8]. Traditionally, water kefir is prepared by the addition of 5–10 % (w/v) of water kefir grains in 5–10 % (w/v) sucrose solution, followed by fermentation at room temperature of 20–25 °C in anaerobic conditions for 24–72 h [33]. Water kefir grains require a nutritious base with high mineral content. For this reason, at the household level utilization of distilled, reverse osmosis, and activated carbon filtered water are not suggested as they contain lower or trace levels of minerals. Besides, the utilization of tap water is not suggested because of the chlorine and fluoride content, as these substances can harm probiotic microorganisms in the grains. Moreover, equipment made from iron, copper, aluminium, or other metals is not suggested to be used since grains are reactive to these metals [35]. When the fermentation is completed, the grains are isolated from kefir by sieving; then, they are washed and dried at room temperature. These isolated grains are stored in a cooling tank for the next fermentation procedure [36]. Before the next water kefir production, the frozen-stored water kefir grain inoculum is thawed and reactivated again during three consecutive pre-fermentations [21]. However, this procedure can be modified according to the desired final product and the duration of the fermentation can be as short as 12 h [37,38]. The final pH is generally measured at about 3.5–4.5 and the alcohol content is below 1 % [33]. After the fermentation at room temperature, the water kefir becomes cloudy, carbonated, and straw-colored [30]. If the water kefir is prepared in the right way, the sucrose in the base solution is expected to be completely converted into carbon dioxide, exopolysaccharides, ethanol, acetic acid, lactic acid, and various other fermentation products including volatile aroma compounds [12,32].

Characteristics of water kefir such as such as its texture, colour, aroma, volatile profile and nutritional and microbial composition can affected by the raw material, production (conditions of fermentation) and storage conditions. Water kefir typically has a slightly viscous texture due to the polysaccharides produced during fermentation. The texture can range from thin and watery to slightly thicker and gel-like, depending on factors such as fermentation time and strain selection [39]. As dried/fresh fruits and vegetables can be used to produce water kefir, phenolic and antioxidant profile of the end product is being affected accordingly [40]. Additionally, use of different fruits (persimmon and mandarin fruits) has been resulted in good organoleptic parameters as well as higher phenolic content in water kefir samples [41]. The volatile compounds produced during fermentation contribute to the aroma and flavour of water kefir. These compounds can include organic acids, esters, alcohols, and other aromatic molecules, which vary depending on the fermentation conditions and ingredients used. friskWater kefir's aroma and flavour are mostly formed by the yeasts such as *Saccharomyces* sp., *Candida* sp., *Kluyveromyces* sp. and *Pichia* sp [6]. Acetic acid, a marker for the metabolic activity of microorganisms in water kefir, contributes to the fruity flavor and aroma characteristic of water kefir, attributed to its esters [41].

**Fig. 2:**
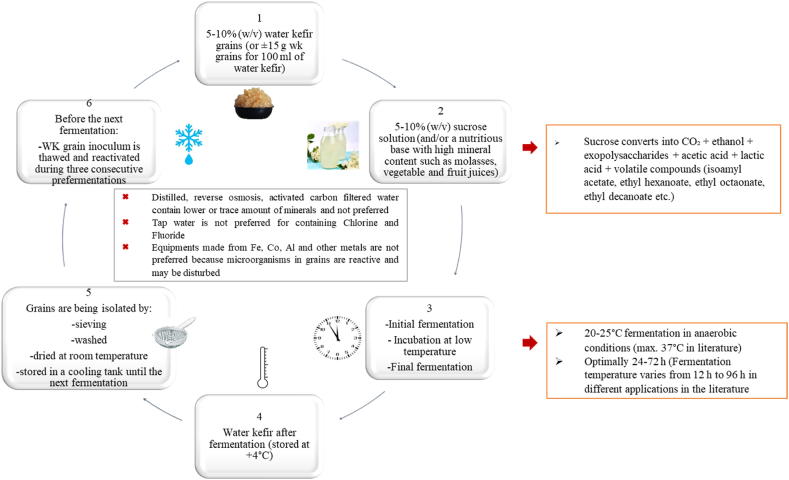
Industrial production flow of the water kefir beverage.

The industrial production of water kefir includes the following steps: initial fermentation, incubation at low temperature, and final fermentation. This process requires 2–4 days of anaerobic fermentation and at the end of the final fermentation, the grains are separated through sieving from the kefir liquor. The grains can be used for the next water kefir production. Taking the aliquot of the grains from the fermented product and then adding it to the new product to be fermented is called backslopping. In the production of 100 mL of water kefir, an average of 15 g grains is used during industrial water kefir production. During backslopping, the starter culture from the previous fermentation is fed every 72 h and during the procedure, the water kefir mass increases up to 600 g [42]. Temperature and other fermentation conditions such as the microbial content of the kefir grain, type of substrate, and duration of the fermentation should strictly be controlled to obtain the desired characteristics of the product [43]. In industrial water kefir beverages, molasses and vegetable or fruit juices can also be used as the base solution [31]. The industrial production flow of water kefir beverages is shown in Fig. 2.

The species dominant in water kefir grain determine the aroma, flavor, and acidity of the final product [8]. In addition, the variety of these species depends on the geographical origin of the grains, the substrate, and the fermentation conditions, as mentioned before, which agrees with the fact that water kefir grains contain different types of dominant bacteria mainly according to their origin [8,18]. In different samples gathered from the UK, USA, and Canada, Actinobacteria, Firmicutes, and Proteobacteria were identified. However, in the samples derived from Germany, Lactobacillaceae were the most abundant, followed by Bifidobacteriaceae. In the samples derived from Turkey, a higher number of *L. kefiranofaciens* was identified [33]. Despite this fact, all the above-mentioned factors can be controlled and/or modified during production to obtain predefined characteristics [8].

Otles and Cagindi (2003) emphasized that the sensory and chemical properties of the final product depend on the type of substrates and microbial composition of the grains used [44]. Chemical characteristics that influence sensory quality and vice versa are also valid. For instance, water kefir with a higher *Ziziphus jujuba* mill sirup concentration (%, v/v) contained higher total soluble solids and was darker than the sample containing a lower concentration of sirup (*p* < 0.05) [45]. It can be generalized as the color of the water kefir changes apparently during fermentation, and the lightness, which is indicated by the L* value, is reported to increase [46,47].

After complete fermentation, the main metabolites are known to be lactic acid and ethanol; however, in lower amounts, acetic acid, glycerol, and mannitol are also produced. In addition, some volatile compounds were found such as ethyl acetate, isoamyl acetate, ethyl hexanoate, ethyl octanoate, and ethyl decanoate [30,48]. Among all the volatile compounds; isoamyl acetate, ethyl hexanoate, ethyl octanoate, and ethyl decanoate were found to have the highest impact on the aroma of the product, which contributes to a fruity floral taste [[46], [47], [48]].

During water kefir fermentation, sugar and total soluble solids (TSS, Brix) concentrations change. Sucrose as the main substrate is hydrolyzed into glucose and fructose by an invertase enzyme produced by yeasts. The TSS content tends to decrease together with the sucrose content [49]. Furthermore, the production of organic acids decreases the pH rapidly, and the concentrations of lactic acid and acetic acid increase after 72 h of fermentation [48]. Generally, water kefir prepared from vegetable juices contains higher amounts of lactic acid and acetic acid in the final products than water kefir prepared from fruit juices [46,47,50]. According to a previous study, both milk and water kefir were fermented using the same kefir grains, and the ethanol concentrations were measured to be higher in the water kefir (up to 2.14 ± 0.12 % w/v), while lactic acid concentrations were lesser (up to 0.16 ± 0.01 % w/v) [26]. On the other hand, the chemical content of water kefir also depends on the type and intensity of the technological processes. For instance, pasteurizing water kefir increased the acetate and propionate content compared with the non-pasteurized samples after 6 h (*p* < 0.001). Butyrate levels were also found to be higher in the pasteurized samples at 24 h, whereas ammonia content was lower in the pasteurized samples at 24 h [51].

Another important component formed during water kefir production is kefiran. Kefiran is a water-soluble polysaccharide that contains equal amounts of glucose and galactose. It is classified as an exopolysaccharide found in kefir grains and was discovered in 1967. Although many other microorganisms are included in the process, *Lactobacillus kefiranofaciens* contributes the most to the formation of kefiran. In the food industry, kefiran is used for its fibrogenic properties, which are important in food packaging. It also improves the viscosity of the products in which it is used. In addition, it is used as a thickener, stabilizer, emulsifier, fat substitute, and gelling agent in the food industry [33]. It also has hydrocolloid and foam-forming effects as well [23]. The foam-forming effect is considered a cause of ethanol and carbon dioxide production due to yeast growth [23]. Kefiran is considered a safe and non-cytotoxic material [33].

Water kefir is preferred mostly by individuals who have allergies, intolerances, or follow specific diets such as vegan diets, and by those who cannot consume dairy products due to religious practices [33,46,47,52,53]. To meet the increased demand for non-dairy fermented beverages and to create more tasty and nutritious beverages, various fruit and vegetable juices (dried figs, grapes, lemons, pomegranates, cabbage, hawthorn, soy, etc.) are used in the technological production of water kefir [33,[54], [55], [56]]. Fermentation of kefir grains with fruit juices releases glutathione, organic acids, and phenolic compounds, all of which are considered antioxidants [33,53]. Black and green tea are used as an alternative to fruit and vegetable juices for the production of water kefir. Herbal teas are rich in bioactive compounds and pigments that are beneficial for health [57]. The main aim of combining water kefir grains with various herbal extracts or juices is to widen the range of market products for the pleasure of consumers. The popularity and consumption of water kefir-based beverages are expected to increase this way [31,46,47]. Even though it is still not as popular as it is expected to be, in some parts of the world, water kefir is considered a traditional beverage. In Latin American countries, pineapple, brown sugar, cinnamon, and kefir grains are used for the production of “tepache”; in Italy “kefir d'uva”; and in the rural areas of Greece, ginger-based water kefir is consumed [37]. However, the independent of the type of product, it has been reported that the overall consumer acceptability for water kefir decreased after storage for 21 days at 4 °C [45].

## 4 Limitations of water kefir for human consumption

The main microorganisms responsible for water kefir fermentation are LAB, AAB, and yeasts [[30], [31], [32]]. Nonetheless, the quantity and types of microorganisms highly vary among samples in relation to their origin [30,58]. These microorganisms combine to form a transparent and elastic structure of 5–20 mm in diameter with linear α-1,6 and α-1,3-linked side bonds [34,59].

The major factor that influences the fermentation process of water kefir production includes the lack of availability of defined culture. Therefore, the most widely used method in practice is the back-sloping method, which employs the use of undefined and complex culture from one fermentation to the subsequent cycles. Several authors have investigated the effect of different cultures on their efficiency in water kefir fermentation and have obtained varied results.

There are some limitations to water kefir consumption, both human consumption and technological processes. Unstable fermentation and low grain growth are the main limitations of the industrial production of water kefir. Re-isolation of the grains is crucial for the next fermentation process [33]. Laureys and De Vuyst (2014) indicated that the grains form 86 % of the water kefir, and during the freezing process, the ice crystals may damage the polysaccharide structure of the grains [48]. This is valid for the thawing process, and this type of damage is generally irreversible, and the structure of the grain cannot be restored during pre-fermentation or production [60]. For this reason, other methods such as freezing fresh water kefir grains in liquid nitrogen before the drying process have been suggested [19]. Another reason for low kefir growth is excessive substrate concentrations, which result in high osmotic stress [60]. In addition, probiotic microorganisms are highly sensitive to physicochemical stresses such as pH, acidity, temperature, and preservatives, and these factors can damage the growth of species [33]. Another limitation is the possible existence of some contaminants that may cause health problems in humans. Improperly prepared or stored water may be contaminated with undesired bacteria, molds, or other pathogens. Contamination can occur during the fermentation process or through inadequate hygiene practices in handling the ingredients or equipment. A recent systematic review screened papers on both milk and water kefir and concluded that kefir beverages are one of the most efficient bioabsorbents for eliminating food contaminants. Furthermore, employing kefir as an anti-contaminant may offer benefits such as high efficacy, affordability, simplicity, and specificity. As a result, kefir bioabsorbents show great promise in reducing food industry contaminants and thereby mitigating potential health risks to humans [61]. Xenobiotic contaminants in water kefir were studied, and water kefir was found to be safe for all contaminants, including alkylphenol, bisphenol, and alkylphenol ethoxylates [62]. The ability of water kefir grains to remove aflatoxin B1 (AFB1), a toxic mycotoxin, under various conditions was studied. Results showed that water kefir grains, dominated by *Lactobacillus, Acetobacter*, and other microorganisms, effectively removed AFB1 across different pH and temperature ranges. Removal was primarily through absorption, with 49.63 % of AFB1 retained after washing. WKG treatment reduced AFB1-induced mutagenicity and decreased AFB1 levels in cow milk and tea soups by over 54 %, indicating their potential as bioabsorbents for detoxification in food and feed [63]. Although the literature does not have serious health risks associated with water kefir, it is highly crucial to point out that quality control measures should be followed. This would be beneficial to avoid/minimize the potential health risk associated with water kefir consumption. Hence, it is significant to follow quality control measures at every point of the food system until the product has reached consumers. Producers should follow proper hygiene practices during the production and storage of water kefir in addition to using high-quality raw food sources.

## 5 Challenges in water kefir production

As mentioned before, water kefir is produced by fermenting a water-based solution including fruits (apple, pear, kiwi, grape, melon, tomato and others), vegetables (onion, ginger, soybean, carrot etc.), and a sugar source using water kefir grains [8]. These grains are composed of approximately 10–14 % of dry matter and the temperature and duration of fermentation typically range between 21 and 30 °C for 4–8 days, respectively [34]. Since there is no defined culture for the production of water kefir, its production has been followed mainly on a home scale or under less aseptic conditions. Numerous factors affect the production parameters of water kefir production, including the source of the substrate, fermentation conditions, non-availability of defined cultures, and metabolite interactions. Despite these efforts, the major challenge is to address the scaling-up issue of water kefir production.

Fresh/dried figs are most commonly used for the production of water kefir. They are considered to produce optimum fermentation compared with other fruits because the former consumes glucose at a faster rate and maintains the optimum rate of production of different metabolites such as lactic acid, acetic acid, carbon dioxide, and ethanol. Approximately 30 various compounds are reported to be generated during fermentation for the production of water kefir. The main factors accruing to the figs are their ability to be growth-promoting factors such as calcium, which can be extracted in cold water, and their ability to withstand moderate heat treatment [64]. Additionally, calcium and higher buffering capacity influence the increase in grain mass because lower pH inhibits the process of fermentation by influencing the activity of LAB glucansucrase, which ultimately results in decreased glucan and grain mass formation [65]. Because dried fig contains higher amounts of calcium than fresh figs and other fruits, fig is believed to be the best source for the production of water kefir. For a similar reason, hard water containing more calcium and magnesium ions is reported to be more suitable for water kefir production. The presence or absence of oxygen also greatly influences the production of water kefir. One of the dominant bacteria found in water kefir is AAB, and its proliferation varies with the increase in grain mass. AAB are believed to grow under both aerobic and anaerobic conditions; however, the former conditions are more conducive to their proliferation. More proliferation of AAB results in the higher production of acetic acid and ethyl acetate and lower production of fruity esters, which drastically reduces the pH, thereby decreasing the growth of water kefir grains. Furthermore, depending on the substrate source, water kefir grain growth varies. Low nutrient concentrations during the production of water kefir result in reduced metabolic activity accompanied by limited oxygen escape and reduced carbon dioxide production. All of these activities are conducive to the proliferation of AAB and result in decreased water kefir growth. Nutrient content also significantly contributes diversity to the growth of fermentative microorganisms [3]. For instance, high nutrient concentrations promote the growth of LAB bacteria, including *Lb. nagelii* and *S. cerevisiae,* whereas low nutrients inhibit the growth of *Lb. hilgardii* and *D. bruxellensis.* Because of the potential effects on which species predominate and subsequent effects on the characteristics of the final beverage in terms of metabolites generated, their concentration, and final beverage flavor and aroma, the substrate used and fermentation conditions should be carefully considered.

Water kefir grains consisting of isolated LAB strains, including *Lb. hilgardii*, *S. cerevisiae*, and *A. tropicalis,* were used and studied for their metabolite interactions [66]. Thus, water kefir produced was reported to possess a gelatinous structure, and the metabolites produced were mainly lactic acid and acetic acid. Fewer attempts have also been made wherein mixed cultures were investigated for the production of “kefir-like beverages” from fruits [47] and vegetable juices [46]. Interestingly, commercial freeze-dried culture has been identified to contain *Lactobacillus*, *Lactococcus*, *Leuconostoc*, and *Saccharomyces* with a count of 10^9^ CFU g^−1^. In addition, scaling up the production of water kefir has always remained the biggest challenge for the industry. This is mainly because of the non-availability of defined and optimized fermentation processes, including defined starter culture, fermentation time-temperature combination, and preservation techniques. These results in the varied quality of water kefir beverages hamper the chance of scale-up at the industrial level. The only report available in the literature related to the scaling-up of water kefir production is a batch fermentation of 6 L [60] however, the reported challenges with this experiment included limited grain growth and unstable fermentation conditions. Further, the grins stored at the deep freezing temperature of approximately −18 to −20 °C irreversibly damaged the structure which could not recover even after the back-slopping method [67]. In addition, industries mainly employ the use of demineralized water, which generally lacks calcium and has low buffering capacity. The use of muslin cloth during the fermentation process also allows the process to be in an aerobic mode, which ultimately results in higher proliferation of AAB, thereby inhibiting the growth of water kefir grains [21].

Although there are some challenges in the production of water kefir, as mentioned above, it has become a popular drink because it is appropriate for diets that are plant-based, vegan, or lactose-free. Different types of food can be used to obtain water kefir, which results in differences in taste and aroma. This allows producers to offer various options to customers and increase their acceptance of water kefir. Market trends have shown that the growing consumption of water kefir is closely linked to increasing consumer awareness of the benefits of fermentation and the expanding availability of probiotics in various food products [6,68]. A study reported that consumer acceptance of water kefir is positive, with 53 % liking the product and 50 % willing to buy [69]. The global kefir market, valued at $1.23 billion in 2019, is forecasted to reach $2.40 billion by 2032 [70].

Some studies have provided novel beverages using water kefir as a base [71]. This study aimed to understand the physicochemical, rheological, and sensory aspects of a whey-enriched carrot juice beverage (carrot juice: whey ratios of 100:0; 95:5; 85:15; 75:25; 65:35) fermented with water kefir or milk starter cultures for 21 days at 4 ± 1 °C. The values of density, pH, and total soluble solids generally decreased with longer storage times for all studied samples. However, as storage times were extended, the amounts of ethanol, total dissolved solids, and degree of fermentation increased. In addition, it was discovered that every model sample displayed pseudoplastic behavior. All samples, regardless of the starting culture utilized, were deemed most acceptable when they included 25 % (% *w/w*) of whey [71]. Koh et al. (2018) explored the possibility of making a novel water kefir beverage from fermented pumpkin. To create a pumpkin-based beverage, the ideal concentrations of pumpkin puree and brown sugar were reported as 22.28 and 9.07 % *w/v*, respectively. The mixture was then fermented for 24 h at 32 °C using water kefir grains. The water kefir beverage made from fermented pumpkin was shown to be alcohol-free and to have great cell viability of yeast- 10^9^, AAB- 10^9^, and *Lactobacillus-* 10^12^ CFU mL^−1^ [72]. Using Russian olives, a novel water kefir product with high antioxidant activity and potential probiotic activities was studied. The optimal conditions for fermentation were found to be an incubation temperature of 31.2 °C, a duration of 24 h, and a concentration of 30 % Russian olive juice, which had the highest total phenolic content, antioxidant activity, and microbiological survival of water kefir microorganisms [73].

In addition to novel approaches toward water kefir beverages, innovative fermentation techniques (strain selection, encapsulation methods to enhance probiotic stability and shelf-life) have been suggested to overcome the challenges associated with water kefir production. The development of a novel kefir culture through the microencapsulation of dominant bacteria in kefir grains has been proposed, offering improved stability and control over fermentation processes [73,74]. This study aimed to encapsulate water kefir microorganisms and bioactive compounds in Russian olive water kefir using spray drying as an encapsulation method, thus developing a symbiotic functional powder. These results were promising in terms of physicochemical and microbial characteristics [74]. Furthermore, the backsloping method has been established for the large-scale production of kefir beverages, significantly increasing production while maintaining microbiological, nutritional, and physicochemical characteristics [75].

## 6 Health aspects of water kefir consumption

Water kefir is known to have various health benefits, including antimicrobial, anti-inflammatory, antiulcerogenic, and antioxidant activities, reduction of oxidative stress, immunomodulation, anticancer, and antiobesity effects, which have been demonstrated in many clinical and preclinical studies [8,11,33,37,51,55,66,[76], [77], [78]]. Water kefir was found to have a protective effect against inflammation-induced intestinal barrier disruption. After water consumption, beneficial short-chain fatty acid (SCFA) production and *Bifidobacterium* species in the gut increased, whereas excess proteolytic fermentation compounds decreased. Pasteurization of water kefir appeared to enhance these mentioned beneficial health effects [51].

Water kefir grains have been reported to demonstrate antimicrobial activity against pathogenic microorganisms such as *Candida albicans, Salmonella typhi, Shigella sonnei, Staphylococcus aureus, Escherichia coli, Listeria monocytogenes, Streptococcus pyogenes, Streptococcus salivarius*, and *Pseudomonas aeruginosa* [31]. This can be achieved through the adhesion of these microorganisms to the intestinal mucosa [37]. In other words, the antimicrobial activity of water kefir is due to the presence of propionic, citric, and malic acids in water kefir, which increases the acidity in the gut and inhibits the growth of pathogens [79]. Silva et al. (2009) demonstrated that water kefir grains obtained at the end of brown sugar fermentation significantly inhibited the reproductive activity of *Candida albicans* after 144 h [80].

The detoxifying effect of water kefir grains on heavy metal ions has been demonstrated in some studies [38,81]. Alsayadi et al. (2013) found that water kefir could reduce the Fe^3+/^ferricyanide complex to the ferrous form [38]. The grains can interact with heavy metal ions dissolved in an aqueous solution, and their metabolic activity seems to depend on the sugar content, contact time, pH, buffer, and kefir grain-to-metal solution ratio. However, these heavy metal ions could be absorbed on the grain surface only in the presence of sucrose. Moreover, it was found that if the initial pH value is too low (3.5), metal ions may remain in the solution. Instead, if the initial pH is too high (6.0), metal ions appear to be quickly adsorbed [81].

Antioxidants are substances that neutralize the negative health effects of free radicals or reactive oxygen species that damage cells [37,38]. Alsayadi et al. (2013) identified the antioxidant activity of water kefir by examining 2,2-phenyl-1-picrylhydrazyl (DPPH) radical-scavenging activity, inhibition of ascorbate auto-oxidation, and reducing power activity. The DPPH-scavenging activity was found as 9.88–63.17 % whereas inhibiting ascorbate oxidation was observed at 6.08–25.57 % [38]. Phenolic acid derivatives and flavonoids in water kefir are thought to be responsible for the antioxidant capacity [16]. Water kefir beverages that have a base of fruit or vegetable juices are known to have enhanced antioxidant activity [37]. Moreover, the scavenging activity of water kefir with fermented fruit juice seems to be 10 % higher than that of kefir prepared with fruit puree [82].

Kefiran has a particularly hypocholesterolemic and hepatoprotective effect on human health [33]. Kefiran's effect on lowering serum cholesterol was supported by a study [83]. Seems like kefiran alone cannot inhibit the absorption of cholesterol in the food itself, but it limits enterohepatic circulating cholesterol in the intestine, and this can prevent hepatic disorders caused by serum cholesterol. It has also been shown to have a positive effect on liver disorders and decrease intestinal histamine and lowering histamine intolerance. It can also be used as a cicatrizing agent for curing some infections because of the increased production of interferon β-cortisol and noradrenaline in human cells [33]. Kefiran can increase the number of *Bifidobacteria* in the colon and improve the immune system [83].

Lactic acid bacteria, which are abundant in water kefir, have hepatoprotective effects in animal models [[84], [85], [86]]. Specifically, *Lb. plantarum* AR501 improves antioxidant status in mice with liver injury. When *Lb. plantarum* AR501 was orally administered to injured mice in vivo, it enhanced their antioxidant state by reducing lipid peroxidation and restoring antioxidant enzyme activity. Meanwhile, the Lb. plantarum AR501 group's nuclear factor erythroid 2-related factor 2 (Nrf2) gene expression was noticeably elevated. This led to the upregulation of multiple antioxidant genes in the liver of mice, including heme oxygenase-1, NAD(P)H:quinone oxidoreductase-l, glutathione S-transferase, and glutamate cysteine ligase [84].

Afifudin (2019) demonstrated the hepatoprotective effects of water kefir. The liver is the most critical organ for lipid metabolism [87]. If the lipid concentration exceeds a certain level, the antioxidant superoxide dismutase (SOD) levels begin to decrease. This antioxidant is known for its hepatoprotective effect and decreases the risk of liver diseases. In this study, a sample of 15 male rats (*Rattus norvegicus*) was fed ad libitum for 8 weeks. Quail egg yolk (5 mL/200 g of body weight) was given to other groups (second and third) for 4 weeks. For the next 4 weeks, the third group was also given 5 mL of orange-based water kefir to each 200 g of body weight. The third group, which was administered orange water kefir, had significantly higher levels of SOD activity in the liver [87]. In another study, the hepatoprotective effect of water kefir in Sprague-Dawley rats was investigated. The rats were divided into four groups and given different doses of water kefir with sugar solution (sugar solution of 1, 2, and 3 mL, respectively) for seven days. Subsequently, a sublethal dose of acetaminophen (640 mg/kg) was administered to rats to create hepatocellular damage. The hepatoprotective effect of water kefir was measured by measuring aspartate aminotransferase (AST) and alanine aminotransferase (ALT) levels. The results showed that water kefir decreased AST and ALT levels significantly (*p* < 0.05) which is responsible for the hepatoprotective effect. Furthermore, the more water kefir was given (3 mL), the higher the AST and ALT-reducing effects. Water kefir is thought to affect AST levels by protecting mitochondria from oxidative stress induced by acetaminophen toxicity and maintaining the integrity of the mitochondrial membrane [88].

Water kefir consumption reduced the proliferation, migration, and invasion of 4T1 cells in mice. Rats with 4T1 cancer cells were administered kefir water for 4 weeks. Kefir water was found to be cytotoxic toward 4T1 cells at IC_50_ (half-maximal inhibitory concentration) of 12.5 and 8.33 mg/mL for 48 and 72 h, respectively. Tumor size and weight (0.9132 ± 0.219 g) were significantly decreased and helper T cells (5-fold) and cytotoxic T cells (7-fold) were increased in the kefir water-treated group. The rate of lung and bone marrow metastases and tumor-related cytokines was also found to be lower in the rat group than in the control group. Furthermore, proinflammatory and proangiogenic markers were significantly reduced in the kefir water-treated group [89]. Soares et al. (2021) confirmed the therapeutic potential of water kefir in breast cancer using an integrative review study [90].

The gastroprotective effects of water kefir on ulcer induction with acidic ethanol were studied in male rats. Pretreatment with water kefir continued for 14 days. Male mice (C57BL/J6) were separated into five groups: the control group received vehicle without ulcer induction; the ulcerated group received vehicle; the lansoprazole group received 30 mg/kg/day lansoprazole; and the water kefir (WK15 and WK30) groups received WK at a dose of 0.15 or 0.30 mL/kg/day, respectively. Gastroprotection was measured by ulcer area, ulcer index, and ulcer reduction percentage. Pretreatment with water kefir had a gastroprotective effect similar to that of lansoprazole. The gastroprotective effect was thought to be a result of decreased oxidative stress due to water kefir consumption. Water kefir consumption also decreased protein oxidation and increased SOD and catalase activity. Water kefir increased the activity of the antioxidant enzyme and prevent gastric lesions induced by acetic ethanol. Water kefir also prevented damage to the gastric mucosa induced by alcohol consumption in animals [76].

Orange-based water kefir's effect on malondialdehyde (MDA) levels in the kidneys of hyperlipidemic rats was investigated. During the research, rats were divided into 3 groups; K+ (positive control group), K (negative control group), and B (interfered group). K+ and B were given quail egg yolk for 4 weeks (5 mL/200 g BW); the K group was fed ad libitum. For the next 4 weeks, the K+ and K groups were only fed ad libitum, and the B group was given water kefir combined with orange juice to each rat as 5 mL/200 g BW (50 % water kefir-50 % orange juice). The hyperlipidemic rat group fed orange water kefir had improved levels of MDA in the kidney tissue. It was concluded that water kefir can protect against renal cell damage, which can cause metabolic disorders [91].

Type 2 diabetes mellitus (T2DM) is a chronic systemic disease that is a global public health problem [92]. Diabetes affects 10.5 % of the adult population (20–79 years old), with nearly half of those affected unaware of the disease. According to The International Diabetes Federation (IDF), one in every eight adults, or roughly 783 million people, will have diabetes by 2045, a 46 % rise [93]. Type 2 diabetes affects more than 90 % of individuals with diabetes, and it is caused by socioeconomic, demographic, environmental, and genetic variables. However, diabetes can be reduced by implementing preventive steps for T2DM and providing early diagnosis and good care for all types of diabetes [93]. Furthermore, it has been shown that water kefir might have a beneficial impact on T2DM [94]. In a previous study, adult Wistar rats were made diabetic through intraperitoneal injection of streptozotocin. One group was given kefir in the water (10–30 % concentration) for 5 weeks. Body weight, serum glucose, and lipid levels were measured. An increase in body weight and improved lipid (total cholesterol, triglycerides, low-density lipoproteins, and very low-density lipoproteins) and glucose profiles were observed in diabetic rats compared with the control group [95]. Another study found that the consumption of Philippine coco water kefir lowers cholesterol levels through the activity of bile salt hydrolase [96]. In a randomized block design study, the antihyperglycemic effect of water kefir against metformin in alloxan-induced diabetic mice was investigated. Alloxan was inducted (65 mg/kg body weight) into 75 adult female and male mice (body weight between 35 and 50 g). Serum glucose levels were recorded at the end of the first hour, and mice with a glucose level above 200 mg/mL were classified as diabetic. Then, the mice were divided into five treatment groups: negative control, positive control, low-dose, medium-dose, and high-dose water kefir consumption. Water kefir consumption significantly lowered serum glucose levels in the group that received medium and high doses in the following 0.5, 1, 2, and 3 h. In the low-dose treatment group, no antihyperglycemic effect was observed [97]. Water kefir was demonstrated to improve the serum and hepatic lipid profiles of rats in another study [98].

Most recently, the antidiabetic potential of *Lb. paracasei* isolated from Malaysian water kefir grains, which has previously been found to have outstanding probiotic qualities and significant antioxidant activities, was assessed. To create a T2DM model, a high-fat diet/streptozotocin induction was performed, followed by treatment with *Lb. paracasei* isolated from water kefir grains. After 14 weeks, groups treated with *Lb. paracasei* showed less insulin intolerance compared with untreated diabetic mice. Furthermore, in diabetic mice, treatment with isolated *Lb. paracasei* from water kefir grains altered the expression of numerous genes involved in glucose homeostasis and lipid metabolism as well as reduced the oxidative stress caused by hyperglycemia [94].

There is less focused scientific evidence on the effects of water kefir consumption on human health. For instance, β-galactosidase is an enzyme that hydrolyzes lactose into glucose and galactose, which could be used in treating lactose intolerance. In particular, coconut water kefir was shown to be a rich source of β-galactosidase-producing microorganisms, which may be useful for treating lactose intolerance [99]. Another possible health benefit of water kefir consumption is its neuroprotective effect. Kumar et al. (2021) found that water kefir showed a neuroprotective ability against H_2_O_2_-induced oxidative stress in differentiated human neuroblastoma (SH-SY5Y) cells [100].

In addition to all the aforementioned health effects, each member of the microbial community of water kefir grains has a specific health effect. *Lb. paracasei,* which was isolated from water kefir, was shown to have antimicrobial, antifungal, and antioxidant effects on human health [11]. *The Lb. rhamnosus* GG strain was found to have an antidiabetic effect. A diet supplemented with *Lactobacillus Mali* APS1, which is isolated from sugary kefir grains, was found to be effective in maintaining blood glucose levels in obese rats [101,102]. *Lacticaseibacillus casei*, *Lb. acidophilus*, and *Bifidobacterium longum* have hypocholesterolemic effects [103]. *Lb. Mali* K8 was tolerant to lower pH degrees such as 2.5 and it was resistant to the damaging effects of bile salts, pepsin, and pancreatin [102]. There is a great variety of other health benefits of the microorganisms existing in water kefir grains, such as immune-modulation, anti-allergenic, anti-obesity, anti-colitis, anti-asthmatic, regulation of T cells, boosting weight loss, and maintaining glucose homeostasis [[104], [105], [106], [107]]. A list of all possible health effects of water kefir consumption is shown in Table 2.

**Table 2:** Possible health effects of the consumption of water kefir.

Constituent/water kefir consumption	Possible health effect(s)	References
Exopolysaccharides	Exopolysaccharides from water kefir grains showed antibacterial activity against *Escherichia coli* and *Staphylococcus aureus*	[108]
Water kefir consumption	Detoxifying agent towards heavy metal ions	[109]
Water kefir consumption	Antimicrobial potency against *Bacillus subtilis, Bacillus pumilus, Staphylococcus aureus, Escherichia coli, Pseudomonas aeruginosa,* and *Candida albicans* species	[56].
*Lentilactobacillus hilgardii, Lacticaseibacillus paracasei, Liquorilactobacillus satsumensis, Lactobacillus helveticus,* and *Lentilactobacillus kefiri*	Potential probiotics, including good survival in acid and bile environments, bile salt hydrolase activity, antioxidant activity, non-cytotoxicity and high adhesion to Caco-2 cells, and a lack of virulence or antimicrobial resistance genes	[58,110]
*Saccharomyces paradoxus* and *Saccharomycodes ludwigii*	Are able to survive under conditions that simulate the gastrointestinal tract, to adhere to mucin in high percentages; excellent antioxidant activity and probiotic potential; sensitive to antimycotics, except for *Saccharomycodes ludwigii*	[111]
Water kefir consumption	Promoted *Bacteroidetes* abundance; increased acetate, propionate, and butyrate concentrations; hydrolyzed glucans from *Liquorilactobacillus satsumensis* have prebiotic properties	[110]
*Lactobacillus mali, Lactobacillus casei, Leuconostoc mesenteroides, Gluconobacter hansenii,* and *Saccharomyces cerevisiae*	Anti-hyperglycaemic effect	[112]
Water kefir consumption	DPPH scavenging activity (9.88–63.17 %); inhibits ascorbate oxidation (6.08–25.57 %); source of natural antioxidants	[38]
Water kefir consumption	Hypoglycemic and hypolipidemic effects	[95]
LAB isolated from Philippine coconut water kefir	Cheap, safe, and efficient probiotics with cholesterol-lowering properties	[96]
Water kefir consumption	Heavy metal reducing capacity	[81]
*Lactobacillus mali* K8	In vitro probiotic potential characterization, antibiotic resistance, hemolytic activity, tolerance to pH 2.5, and resistance to bile salts, pepsin, and pancreatin, comparable to that of *Lactobacillus rhamnosus* GG ATCC 53103 (reference strain).	[102]
*Lactobacillus mali* APS1	Ameliorated hepatic steatosis by modulating lipid metabolism and antioxidant activity via manipulating specific NAFLD-associated gut microbiota in vivo	[105]
Water kefir consumption	Improved lipid profile	[98]
Orange water kefir consumption	Significantly increased the superoxide dismutase activity in the liver tissue of hyperlipidemic rats	[87]
Orange water kefir consumption	Improved malone dialdehyde level in kidney tissue of the hyperlipidemic rat model, protective against renal cell damage which can lead to metabolic disorder.	[91]
Water kefir consumption	Increased antioxidant enzyme activity, preventing gastric lesions against HCl/ethanol ulcer model by maintaining antioxidant performance in gastric tissue	[113]
Water kefir consumption	Antioxidant capacity	[95]
Water kefir consumption	Hypocholesterolemic and hepatoprotective activities, fermentation operated by kefir grains on fruit releases glutathione, organic acids, and phenolic compounds, all of which act as antioxidants	[33]
Water kefir consumption	Anti-oxidant, anti-apoptosis, and neuroprotective effects are mediated via the upregulation of superoxide dismutase and catalase, as well as the modulation of apoptotic genes (Tp73, Bax, and Bcl-2).	[100,113]
Water kefir consumption	Preventive effect and utilization in the treatment of acute liver failure caused by acetaminophen	[114]
*Lactobacillus* sp.	Probiotic effect	[16]
Water kefir consumption	Increased beneficial short-chain fatty acid production at the microbial level, reduced detrimental proteolytic fermentation compounds and increased *Bifidobacterium genus* abundance -observed benefits increase by pasteurization, pasteurized products improve inflammation-induced intestinal epithelial barrier disruption and increase IL-10 and IL-1β compared to the control group	[51]
Water kefir consumption	Gastroprotection against HCl/ethanol-induced ulcers much like the pretreatment with lansoprazole, decreased protein oxidation while increasing superoxide dismutase and catalase activity, increased antioxidant enzymes	[76]
Water kefir consumption	Antibacterial, anti-inflammatory, anti-diabetic, anti-hyperlipidemic, anti-ulcerogenic, and antioxidant effects	[34,77,115]

In addition to potential health effects of water kefir, regulations related to water kefir are crucial to be pointed out. Global regulations of fermented foods and beverages including Codex Alimentarius Standards have been comprehensively covered in the literature [116]. As water kefir is a fermented beverage, there are different regulations according to the country. In the European Union, water kefir is marketed as a probiotic product under food safety regulations, even though it is not explicitly listed in the food codex [6]. In the United States, milk kefir is listed under “*Code of Federal Regulations, Title 21: Food and Drugs*” [117]. In Australia, water kefir is regulated under “*Standard 2.6.2 Non-alcoholic beverages and brewed soft drinks, classified within the Brewed soft drink category*”. According to this standard, the product must be produced through a fermentation process using water, sugar, and one or more extracts or infusions of fruits or vegetables. Additionally, it must not contain more than 1.15 % alcohol by volume [118].

## 7 Conclusion

Water kefir is a fermented beverage with a unique composition and microbial diversity. Unlike milk kefir, water kefir is produced from water kefir grains and various substrates, including sugar, fruits, and molasses. The microbial composition of water kefir includes LAB, yeasts, and AAB and depends on many parameters such as origin, substrate, and fermentation conditions. Lactobacillus species, particularly *Lb. hilgardii* and *Lb. nagelii*, are commonly found in water kefir grains which contribute to the aroma, flavour, and acidity of the final product. The choice of substrate influences the microbial community and chemical properties of water kefir. The production process, including fermentation time and temperature, also affects microbial diversity. Water kefir offers a viable alternative to dairy-based fermented beverages for individuals with allergies, intolerances, or specific dietary preferences and has been associated with various health benefits, including immunomodulatory and hepatoprotective activities. However, there are some challenges in water kefir production, such as unstable fermentation, low grain growth, and the sensitivity of probiotic microorganisms to physicochemical stresses. Contaminants and improper handling can also pose health risks. Overcoming these challenges requires careful control of fermentation conditions, proper handling and storage of grains as well as adherence to quality and safety standards. Future research needs could include investigations into optimized fermentation processes, development of defined cultures, and standardization of production methods to ensure consistent quality of water kefir beverages. Furthermore, using culture-independent methods like next-generation sequencing can provide a deeper understanding of the microbial composition of water kefir and its potential applications in health and nutrition in addition to technological advancements to overcome problems regarding the production of water kefir. Lastly, since there is a big gap in human studies, well designed human trials could explore the mechanisms behind the observed health benefits of water kefir and its potential applications in various health conditions.

## Data availability statement

The data that was collected and analyzed during this study is contained in this published article and the data that was used to support the findings of this review are listed in the references at the end of the article.

## Funding

The publication of this article was funded by the Open Access Fund of 10.13039/501100001664Leibniz Universität Hannover.

## CRediT authorship contribution statement

**Eda Bozkir:** Writing – review & editing, Writing – original draft, Conceptualization. **Birsen Yilmaz:** Writing – review & editing, Writing – original draft, Conceptualization. **Heena Sharma:** Writing – review & editing, Writing – original draft, Conceptualization. **Tuba Esatbeyoglu:** Writing – review & editing, Writing – original draft, Supervision, Conceptualization. **Fatih Ozogul:** Writing – review & editing, Writing – original draft, Supervision, Conceptualization.

## Declaration of competing interest

The authors declare that they have no known competing financial interests or personal relationships that could have appeared to influence the work reported in this paper.
